# Effect of industrial wastewater on wheat germination, growth, yield, nutrients and bioaccumulation of lead

**DOI:** 10.1038/s41598-020-68208-7

**Published:** 2020-07-09

**Authors:** Amina Kanwal, Muhammad Farhan, Faiza Sharif, Muhammad Umar Hayyat, Laila Shahzad, Gul Zareen Ghafoor

**Affiliations:** 1 Department of Botany, Government College Women University, Sialkot, Pakistan; 2 Sustainable Development Study Center, Government College University, Lahore, Pakistan

**Keywords:** Ecology, Environmental impact

## Abstract

Due to fresh water scarcity, farmers are using polluted water for irrigation. This research was conducted to study the bioaccumulation of Pb in wheat (Cv. Shafaq-2006). The experiment was comprised of seven treatments of lead i.e. 0–1,000 mg Pb/kg. The results revealed that lead severely reduces germination (− 30%), seedling fresh weight (− 74%), seedling dry weight (− 77%), vigor index (− 89%), tolerance index (− 84%), plant height (− 33%), number of leaves (− 41%), root fresh weight (− 50%), shoot fresh weight (− 62%), root dry weight (− 63%), shoot dry weight (− 71%), and root length (− 45%). The physiological parameters also respond negatively like stomatal conductance (− 82%), transpiration rate (− 72%) and photosynthetic rate (− 74%). Similarly, biochemical parameters also showed negative impacts, like carotenoids (− 41), total chlorophyll (− 43), chlorophyll *a* (− 42) and chlorophyll *b* (− 53). Yield parameters like the number of seed/plant, seed weight/plant, 1,000 seed weight and harvest index were reduced by 90%, 88%, 44% and 61%, respectively in T_6_. In addition, protein contents (− 81%), phosphorous (− 60%) and potassium (− 55%) were highly effected in the highest lead concentration (T_6_). Lead accumulation was extremely higher in seeds (119%) as compared to control plants. Lead bio-accumulation above threshold concentrations in crop parts is a serious human health concern.

## Introduction

Fresh water scarcity is a major issue for agriculture in developing countries including Pakistan. To meet water requirements, farmers are now using industrial wastewater^1^. These industrial wastewaters have the high amount of toxic heavy metals (HM) like Pb, Ni, Cd, Zn, Fe, Mn, etc. Heavy metals tend to bio-accumulate in crops and are creating serious health threats to human and ecosystem^2^. Other sources of metal entry in the ecosystem include mining activities, industrial effluents, agronomic practices and use of sludge as manure^3–5^. Worldwide environmental and human health problems are increasing sharply due to heavy metal contamination. Cultivation of crops near these contaminated sites result heavy metal bio-accumulation in crops and poor growth. These accumulated metals pose high risk to humans as well as to livestock health, in case of ingestion^6^.

Zajac et al., investigated 195 TSIP (Toxic Site Identification Program) sites in 33 LMICs (low- and middle-income countries) and reported the 820,000 womoen of childbearing age are at the risk for lead exposure^7^. Lead is a major pollutant in the ecosystem, being persistent/high retention time (150–1,500 years) and shows negative impacts on human^8,9^. Lead can accumulate in plants from various routes including water, air and soil. Prominent harmful effects of Pb include interference with nutrient uptake, germination reduction, reduced photosynthesis, delay in plant growth, disturbed respiration, metabolism alteration, changes in enzymatic activities, changes in root morphology and inhibition of mitosis (in tip)^10–13^, enlarged vacuoles, deformed nucleolus, increased plasmolysis and damaged thylakoid^14^. Heavy metals are also responsible for ROS^5,15^ (reactive oxygen species), MDA (malondialdehyde) formation of excessive amount, mainly in chloroplast and mitochondria of leaves, root and shoot^16^. Rafaqat at el, reported the damage in root ultra-structures in *Brassica napus* L. due to chromium toxicity. They also alter the natural antioxidant enzymes of plants^17^. These ROS have the tendency to attack biomolecules and may cause cell death. Thus, lead contamination serves as a severe problem for agriculture for agriculture^18^.

Rice, wheat, cotton and sugarcane are most important cash crops in Pakistan and account 75% of total output. Wheat is Pakistan's main food crop. Due to the shortage of irrigation water farmers are using industrial wastewater for irrigation. The presence of lead in the food chain can cause chronic health problems^19,20^. The application of plant growth regulator like 5-aminolevulinic acid (ALA) improves metal tolerance in crops^15^. Therefore, this study was designed and executed to investigate the effect of Pb on germination, seedling growth, physiological, biochemical, mineral contents and yield.

## Material and methods

### Germination experiment

Wheat cultivar (Shafaq-2006) was selected as test variety. The Germination experiment was setup in petri dishes (90 mm) in the growth room of The Botany Department, GC University Lahore, Pakistan. Petri dishes were washed, sterilized and blotting paper was placed in each petri dish. Twenty five, seeds were surface sterilized and placed in each petri dish. For irrigation purpose, seven treatments of lead were prepared using lead nitrate.

**Table:** 

Crop specie	Sees per petri dish	Lead treatments	Replicates
1	25	7	4

Treatments were named T_o_, T_1_, T_2_, T_3_, T_4_, T_5_, T_6_ having 0, 100, 200, 400, 600, 800 and 1,000 mg of Pb/L. All the petri dishes were arranged in completely randomized design (CRD). 2 ml of each treatment was added in respective petridish on daily bases. Germination was noted with the emergence of radical. Data was collected for germination %, seedling length, plumule length, radical length and fresh biomass. Seedling vigor index and tolerance index was estimated by multiplying seedling length and germination percentage^21^. The Metals Tolerance Index is a diagnostic metric used to identify samples with a high degree of organisms tolerant of metals.

### Pot experiment

The pot experiment was set up in Botanical Garden (located at The Mall road) of Government College University in ambient condition. No additional instrument was used to control any abiotic factor (light, temperature, humidity, etc.). Each pot (earthen, 12 inch diameter) was washed, dried, lined with polythene bag and filled with 5.5 kg soil (1:6, humus: garden soil) mix. Lead nitrate was added in the soil in different concentration. Treatments were named T_o_, T_1_, T_2_, T_3_, T_4_, T_5_, T_6_ having 0, 100, 200, 400, 600, 800 and 1,000 mg of Pb kg^−1^. Pot’s positions were changed on a weekly basis to keep climatic condition uniform. Data of weather conditions and other meteorological parameters is given in Table S1. All other agronomic practices were kept same for all treatments. All parameters were measured at harvest. After harvesting, plants were washed, dried and stored in polythene zipper bags. Biomass (fresh and dry) of root/shoot and length were measured at harvest using electric balance and measuring rod. For measuring dry weight, crop samples were dried (at 70 °C) for 24 h. Yield attributes were calculated by the protocols of Zadoks^22^.

### Physiological and biochemical attributes

IRGA, ‘Infra-Red Gas Analyzer’ (LCA4 Model) was used to measure stomatal conductance, transpiration and photosynthesis rate. Three leaves from each pot were selected and readings were noted at 75th and 130th day^23^. Chlorophyll contents were determined by following the protocols of Arnon^24^. Carotenoid extraction was carried out in dim light where 1 g of randomly selected fresh leaves was crushed in acetone. Homogenate was filtered and more acetone was added to make the final volume up to 50 ml. Carotenoids were estimated with the help of spectrophotometer at the wavelength of 440.5 nm^25^.

$$``{\text{Carotenoids}}\;{\text{contents}} = \left[ {{\text{V}} \times {383} \times \left( {{\text{As}}-{\text{Ab}}} \right)} \right]{ /}\left( {{1}00 \times {\text{W}}} \right)\text{''}$$

where: “V” is the volume used for analysis; “383” is the extinction coefficient of carotenoids; “As” is the absorbance of the sample; “Ab” is the cuvette error; “W” is the weight of the sample in gram.

### Mineral contents and quality attributes

Potassium was estimated through flame photometer (PF Pt JENWWAY, England)^26^. For phosphorous quantification, 1 g of plant sample was dried, crushed, and placed at 650 °C in the furnace for 3 h^27^. Burned samples was added in 10 ml of H_2_SO_4_ (0.7 N), stayed for about 1 h and later filtered (through Whatman-No-42) and the final volume was raised to 50 ml.

Over dried potassium phosphate (0.43 g) was dissolved in one liter of distilled water to make stoke solution. Different standards (ranging from 2 to 50 ppm) were prepared from this stock solution. Ammonium vanadomolybdate (10 ml) and the respective standard (5 ml) were mixed and stayed for 10 min. Absorbance was noted by spectrophotometer at 410 nm. The same protocol was used for samples. The standard graph was plotted using absorbance value of the standard and absorbance value of phosphorous.

Nitrogen was determined by using Kjeldahl method using the following formula:

$$``{\text{N}}\;{\text{contents}}\left( \% \right) = \frac{{\left( {{\text{A}} - {\text{B}}} \right) \times {\text{Normality}}\,{\text{of}}\;{\text{acid}} \times {14}.0{1} \times {1}0 \times 100\text{''}}}{{{\text{Volume}}\;{\text{of}}\;{\text{sample}}}}$$

where ‘A’ is the HCl used, B is HCl used for blank; 14.01 is atomic mass of nitrogen.

While protein was estimated by multiplying the nitrogen contents (%) with 6.25.

### Lead accumulation

Lead accumulation in roots, shoots and leaves were determined by following protocol of Chandra^28^. Crop samples were 1st washed with double distilled water and later with calcium chloride (10 mM solution). Samples were dried and turned into ashes at 450 °C. Digestion of ash was carried out in HNO_3_ (2%), which was later filtered with glass fiber filter^29^. Lead was quantified by “inductively coupled plasma spectrophotometer (Thermo Electron; Model IRIS Intrepid II XDL, USA)”. Detection limit ranges from 8 to 80 ng L^−1^.

### Data analysis

Results were evaluated through ANOVA and DMR using Costat version 3.03^30^.

## Result and discussion

**Figure 1:**
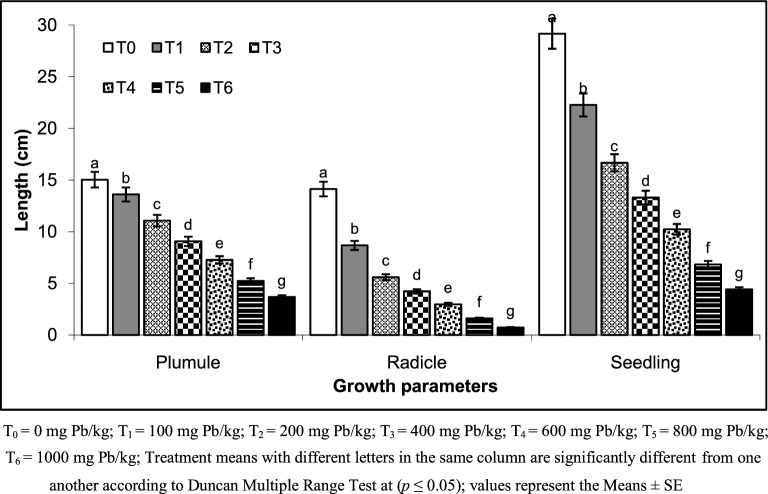
Lead toxicity during wheat germination.

This study has given significant results regarding the Pb toxicity in the germination, growth, physiological, biochemical and yield attributes of wheat. Data regarding germination attributes revealed that Pb treatments significantly affected the germination percentage, fresh weight, dry weight, plumule length, radical length and seedling length of the wheat (Table 1 and Fig. 1). Increase Pb concentration results in negative impacts in wheat. The highest decline recorded was in germination i.e. 30%. Radical length was decreased by 45%, fresh weight of seedling by 74%, dry weight of seedlings by 77%), seedling vigor index by 89% and tolerance index by 74% in T_6_ (1,000 mg Pb/L) compared to the control (Table 1 and Fig. 1). Chun^31^ observed that at 0–0.5 mg Pb/kg non-significant adverse impact of lead on germination index and energy required during the process of germination. Likewise, growth rate of roots and shoots also remain unaffected at low level contamination of lead^32^. However, concentration above 4.5 mg/kg of soil is toxic to plant health, which decreases germination^33^. Roots of plants are more affected by lead toxicity as compared to other plant parts^12,34^. Excessive Pb contamination (including Pb) results in the formation of ‘reactive oxygen species’ (ROS) in mitochondria, chloroplast and cellular compartments^14,35^. The results of (seedling growth) present study are in line with that of Yadav^36^ who stated that growth attributes were significantly affected by increasing the Pb concentration. Co-cropping of *T. minuta* and soybean enhanced the Pb accumulation in *T. minuta* without posing any health risk from grain consumption of soybean^9^.

**Table 1:** Effect of lead toxicity on germination parameters of wheat (*Tritium aestivum* L.).

Treatments	%Germination	Seedling fresh weight (g)	Seedling dry weight (g)	Seedling vigor index	Tolerance index
T_0_	100 ± 0.01 a	0.125 ± 0.013 a	0.064 ± 0.006 a	2,916.67 ± 100.66 a	99.99 ± 3.45 a
T1	100 ± 0.01 a	0.111 ± 0.012 a	0.052 ± 0.010 b	2,226.67 ± 41.63 b	76.33 ± 1.43 b
T_2_	98 ± 0.58 b	0.084 ± 0.006 b	0.043 ± 0.002 c	1638.87 ± 39.40 c	57.14 ± 1.93 c
T_3_	96 ± 0.58 c	0.062 ± 0.016 c	0.030 ± 0.002 d	1,272.40 ± 73.74 d	47.59 ± 2.59 d
T_4_	87 ± 0.62 d	0.025 ± 0.010 c	0.024 ± 0.003 de	893.87 ± 73.60 e	35.08 ± 2.79 e
T_5_	80 ± 0.02 e	0.043 ± 0.003 cd	0.021 ± 0.002 de	546.67 ± 25.72 f	23.43 ± 1.10 f
T_6_	70 ± 0.23 f	0.033 ± 0.006 d	0.017 ± 0.001 e	308.00 ± 38.97 g	15.08 ± 1.91 g
F-ratio	1.0868	35.0304	38.9816	693.2351	539.2539
Significant level	ns	***	***	***	***
LSD (*p* ≤ 0.05)	343.9316	1.01786	0.0085	107.5930	3.9219

T_0_ = 0 mg Pb/kg; T_1_ = 100 mg Pb/kg; T_2_ = 200 mg Pb/kg; T_3_ = 400 mg Pb/kg; T_4_ = 600 mg Pb/kg; T_5_ = 800 mg Pb/kg; T_6_ = 1,000 mg Pb/kg; Treatment means with different letters in the same column are significantly different from one another according to Duncan Multiple Range Test at (*p* ≤ 0.05); values represent the Means ± SE.

Data regarding the growth attributes revealed that Pb significantly (*p* ≤ 0.01; 0.05) affect the growth attributes (Table 2). Minimum plant height (58.2 ± 0.32 cm), root length (13.5 ± 0.24 cm), number of leaves (21.67 ± 0.05) were noticed in T_6_. Dry weight and fresh weight was also affected negatively at higher lead concentration. Minimum fresh weight of roots (0.66 ± 0.05 g), shoot (2.53 ± 0.04 g), and dry weight of roots (1.61 ± 0.02 g), shoot (0.34 ± 0.05 g) were noticed in T_6_ (Table 2). Gopal and Rizvi^37^ also observed that due to increase in lead concentration length and weight of root and shoot decreases. Adverse effects of lead were not apparent at early stage of the plant’s life but detrimental effects increased with the increase in maturity level of plant. Metal uptake, translocation and bio-accumulation are age dependent. Antoniadis^38^ also reported that root development in excessive Pb concentration was poor. The major reason behind the reduction in biomass of plants is reduction in the rate of photosynthesis and nitrogen metabolism^39^. Osma^40^ reported that specie and Pb concentration significantly effect the fresh weight in brassica. Decrease in fresh weight of shoots among different species was observed in following order, *B. juncea.* < *B. oleracea I* < *B. carinata*. Plants may develop resistance and as a result may survive in lead contaminated soils^41^. Gill et al., reported the damage in leaf mesophyll, root tips and disruption of golgbodies, diffused cell wall due to chromium toxicity at 400 µM^14^.

**Table 2:** Effect of various concentrations of lead on growth attributes of wheat.

Treatments	Plant height (cm)	Root length (cm)	Number of leaves	Fresh weight (g)		Dry weight (g)	
				Shoot	Root	Shoot	Root
T_0_	68.7 ± 0.25 a	25.0 ± 0.15 a	26.33 ± 0.58 a	7.02 ± 0.09 a	1.26 ± 0.05 a	5.62 ± 0.01 a	0.92 ± 0.04 a
T1	66.1 ± 0.35 b	22.5 ± 0.26 b	25.67 ± 0.57 a	5.77 ± 0.04 b	1.19 ± 0.03 b	4.45 ± 0.06 b	0.83 ± 0.04 b
T_2_	64.7 ± 0.38 c	20.9 ± 0.25 c	24.33 ± 0.09 b	5.09 ± 0.08 c	1.10 ± 0.09 c	3.79 ± 0.09 c	0.72 ± 0.05 c
T_3_	62.0 ± 0.23 d	18.1 ± 0.38 d	23.33 ± 0.33 c	4.18 ± 0.07 d	0.95 ± 0.03 d	3.25 ± 0.02 d	0.63 ± 0.04 d
T_4_	61.2 ± 0.31 e	16.0 ± 0.52 e	23.00 ± 0.09 cd	3.85 ± 0.05 e	0.86 ± 0.01 e	2.89 ± 0.03 e	0.52 ± 0.06 e
T_5_	60.2 ± 0.25 f	14.7 ± 0.26 f	22.03 ± 0.04 de	3.59 ± 0.06 f	0.78 ± 0.05 f	2.38 ± 0.07 f	0.40 ± 0.02 f
T_6_	58.2 ± 0.32 g	13.5 ± 0.24 g	21.67 ± 0.05 e	2.53 ± 0.04 g	0.66 ± 0.05 g	1.61 ± 0.02 g	0.34 ± 0.05 g
F-ratio	437.63	561.35	31.05	1596.57	59.59	2052.58	76.79
Significant level	***	***	***	***	***	***	***
LSD (*p* ≤ 0.05)	0.5295	0.5479	0.9361	0.1140	0.0872	0.0899	0.07463

T_0_ = 0 mg Pb/kg; T_1_ = 100 mg Pb/kg; T_2_ = 200 mg Pb/kg; T_3_ = 400 mg Pb/kg; T_4_ = 600 mg Pb/kg; T_5_ = 800 mg Pb/kg; T_6_ = 1,000 mg Pb/kg; Treatment means with different letters in the same column are significantly different from one another according to Duncan Multiple Range Test at (*p* ≤ 0.05); values represent the Means ± SE.

**Figure 2:**
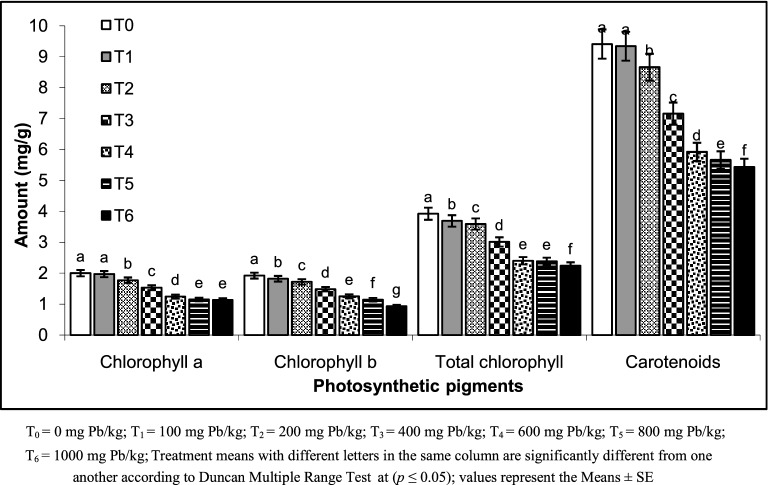
Impact of lead on biochemical attributes of wheat.

Maximum reduction in all biochemical and physiological attributes was noticed in those pots whose soil was spiked with 1,000 mg Pb/kg. all the treatments differ significantly (*p* ≤ 0.01; 0.05). A linear decrease in the physiological and growth attributes was noted with increase in Pb concentration. Regarding the physiological attributes maximum reduction in photosynthetic rate (44%), stomatal conductance (82%) and transpiration rate (72%) was noticed in T_6_ (Table 3). Maximum reduction in chlorophyll *a* (44%)*,* chlorophyll *b* (53%)*,* carotenoids contents (42%) and total chlorophyll contents (43%) was noticed in those pots whose soil was spiked with the 1,000 mg Pb/kg (Fig. 2). Similar results were observed by Zeng^42^ that performed an experimental analysis by applying lead acetate at six different levels (0–900 mg/kg). This may be due to non-toxic effect of lead at low concentration. All the physiological and biochemical attributes of the wheat was reduced by increasing the Pb concentration that might be due to lead toxicity as it damages the chloroplast structure, decreases chlorophyll synthesis, restricted enzymatic and carotenoids activities. Lead is also responsible for obstructions in electron transport chain, deficiency of carbon dioxide (due to closure of stomata) and altered thylokoid membrane. Qadri^43^ reported that metal presence reduce the chlorophyllase and results in low chlorophyll content. Studies have indicated that chlorophyll *b* contents is more disturbed then chlorophyll *a*^39^. Rafaqat et al., reported the decline in photosynthetic rate in *Brassica napus* L. due to the elevated stress of chromium^5^.

**Table 3:** Impact of lead on physiological attributes of wheat.

Treatments	Photosynthetic rate (µmol CO_2_ m^−2^ s^−1^)	Transpiration rate (mmol m^−2^ s^−1^)	Stomatal conductance (mmol H_2_O m^−2^ s^−1^)
T_0_	8.088 ± 0.162 a	0.99 ± 0.020 a	0.428 ± 0.009 a
T1	7.087 ± 0.156 b	0.734 ± 0.016 b	0.324 ± 0.007 b
T_2_	6.787 ± 0.129 c	0.689 ± 0.013 c	0.281 ± 0.005 c
T_3_	5.987 ± 0.132 d	0.654 ± 0.014 d	0.141 ± 0.003 d
T_4_	4.203 ± 0.084 e	0.572 ± 0.011 e	0.127 ± 0.003 e
T_5_	3.502 ± 0.063 f	0.352 ± 0.006 f	0.093 ± 0.002 f
T_6_	2.115 ± 0.036 g	0.263 ± 0.004 g	0.074 ± 0.001 g
F-ratio	4,962.6695	1,830.3037	1,338.3403
Significant level	***	***	***
LSD (*p* ≤ 0.05)	0.09328	0.0173	0.0112

T_0_ = 0 mg Pb/kg; T_1_ = 100 mg Pb/kg; T_2_ = 200 mg Pb/kg; T_3_ = 400 mg Pb/kg; T_4_ = 600 mg Pb/kg; T_5_ = 800 mg Pb/kg; T_6_ = 1,000 mg Pb/kg; Treatment means with different letters in the same column are significantly different from one another according to Duncan Multiple Range Test at (*p* ≤ 0.05); values represent the Means ± SE.

All the Pb treatments significantly (*p* ≤ 0.01; 0.05) decrease the mineral contents and the quality attributes of the wheat (Table 4). Phosphorous content decreased from 11 to 58%, potassium contents from 14 to 34%, nitrogen contents from 38 to 82% and proteins contents from 18 to 81% in the T_6_ as compared to the control. Range of all the yield attributes was as follows, phosphorous contents (18.85–7.51 ppm), potassium contents (409.20–269.57 ppm), nitrogen contents (1.54–0.28%) and protein contents (9.63–1.75%).

**Table 4:** Effect of various concentrations of lead on the mineral contents and quality attributes of wheat plant.

Treatments	Phosphorous contents (ppm)	Potassium contents (ppm)	Nitrogen contents (%)	Protein contents (%)
T_0_	18.85 ± 0.08 a	409.20 ± 0.10 a	1.54 ± 0.01 a	9.63 ± 0.06 a
T1	16.87 ± 0.08 b	350.37 ± 0.15 b	1.26 ± 0.01 b	7.90 ± 0.08 b
T_2_	15.10 ± 0.15 c	349.60 ± 0.30 c	0.91 ± 0.02 c	5.68 ± 0.10 c
T_3_	12.09 ± 0.22 d	339.33 ± 0.21 d	0.80 ± 0.00 d	5.01 ± 0.01 d
T_4_	10.86 ± 0.12 e	311.96 ± 0.12 e	0.55 ± 0.02 e	3.44 ± 0.09 e
T_5_	8.39 ± 0.06 f	291.33 ± 0.21 f	0.41 ± 0.01 f	2.55 ± 0.09 f
T_6_	7.51 ± 0.17 g	269.57 ± 0.31 g	0.28 ± 0.01 g	1.75 ± 0.01 g
F-ratio	2,947.2309	139,098.1018	3,851.3114	4,585.5065
Significant level	***	***	***	***
LSD (*p* ≤ 0.05)	0.2395	0.3729	0.0224	0.1283

T_0_ = 0 mg Pb/kg; T_1_ = 100 mg Pb/kg; T_2_ = 200 mg Pb/kg; T_3_ = 400 mg Pb/kg; T_4_ = 600 mg Pb/kg; T_5_ = 800 mg Pb/kg; T_6_ = 1,000 mg Pb/kg; Treatment means with different letters in the same column are significantly different from one another according to Duncan Multiple Range Test at (*p* ≤ 0.05); values represent the Means ± SE.

Result of Pot experiment were significantly changed by Pb treatments (Table 5). At higher concentration the yield decrease. Minimum number of seeds per plant (16.33 ± 0.58), seed weight per plant (0.85 ± 0.09 g), 1,000 seed’s weight (30.03 ± 0.63 g), straw weight per plant (2.62 ± 0.12 g), and harvest index (263.39 ± 11.52%) was noticed in T_6_ (Table 5). In order to feed the growing population, farmers demand those varieties of crops which give more yields all the yield attributes were reduced by the increasing Pb concentrations. Scientists are also focused on developing new and improved varieties (with genetic engineering), to produce more yield. The current research investigate severe negative impacts of Pb on wheat yield. Xiong^44^ reported that cadmium, lead and zinc accumulate in cabbage and spinach which were grown near smelter. Lead interferes with active sites of enzymes and results in low yield. Zheljazkov and Nielsen^45^ revealed that a 400 m away source of lead (through air) affected corn mint yield by 16%. Similarly, 14% decrease in yield of essential oils was observed due to air pollution^46^. Gill et al ., reported increased size and number of starch grains due to chromium in oilseed rape cultivars^24^. The presence of heavy metals revealed elite molecular transporters (MTs) genes that were responsible for water transmembrane transporter activity^5^.

**Table 5:** Effect of various concentrations of lead on yield attributes of wheat.

Treatments	Number of seed/plant	Seed weight/plant (g)	1,000 seed weight (g)	Straw weight/plant (g)	Harvest index (%)
T_0_	158.68 ± 28.04 a	7.57 ± 1.10 a	54.60 ± 0.79 a	6.98 ± 0.13 a	700.29 ± 12.99 a
T1	84.33 ± 18.01 a	4.54 ± 1.22 b	46.39 ± 0.48 b	5.13 ± 0.08 b	515.41 ± 8.04 b
T_2_	55.67 ± 19.86 ab	3.01 ± 1.07 c	43.63 ± 0.33 c	4.48 ± 0.05 c	449.56 ± 5.50 c
T_3_	37.00 ± 16.52 b	2.28 ± 0.98 cd	42.79 ± 0.14 c	4.07 ± 0.06 d	408.39 ± 6.10 d
T_4_	21.00 ± 1.00 b	1.49 ± 0.06 cd	41.48 ± 0.19 d	3.69 ± 0.15 e	370.65 ± 14.94 e
T_5_	19.00 ± 1.00 b	1.34 ± 0.03 d	39.11 ± 0.79 e	3.32 ± 0.08 f	333.84 ± 8.63 f
T_6_	16.33 ± 0.58 b	0.85 ± 0.09 d	30.03 ± 0.63 f	2.62 ± 0.12 g	263.39 ± 11.52 g
F-ratio	3.3286	24.2617	557.7549	577.7730	684.2652
Significant level	*	***	***	***	***
LSD (*p* ≤ 0.05)	104.6137	1.4529	0.9465	0.17924	39.2345

T_0_ = 0 mg Pb/kg; T_1_ = 100 mg Pb/kg; T_2_ = 200 mg Pb/kg; T_3_ = 400 mg Pb/kg; T_4_ = 600 mg Pb/kg; T_5_ = 800 mg Pb/kg; T_6_ = 1,000 mg Pb/kg; Treatment means with different letters in the same column are significantly different from one another according to Duncan Multiple Range Test at (*p* ≤ 0.05); values represent the Means ± SE.

The results of the Pb accumulation in wheat plant including root, shoot and seed were depicted in the (Table 6). Significant variation in the treatment means was revealed. The extent of increase in lead concentration was quite alarming compared to control treatments. For instance, roots showed 4,600% enhance in Pb quantity in T_6_. Likewise, shoots and seeds showed an increase of 9,800% and 118% in Pb concentration, respectively. Lead concentration decreases in parts of the wheat in the following order root > shoot > seeds. The considerable increase in lead concentration was noticed in T_6_ as compared to the control (Table 6). Lead uptake by roots is directly proportional to the lead concentration. Other factors which contribute towards Pb adsorption include: type of fertilizer, amount of fertilizer, microbial activity, soil pH, precipitates of carbonate/phosphate and concentration of extractable lead in soil^47^. Antoniadis^38^ reported lead translocation decreases significantly as the metal moves away from root, which implies that roots accumulate more lead then seeds and shoots. Different studies revealed high accumulation rate of lead in roots; Kenaf root may retain upto 85% of total Pb present in plant body^48^, *Thlaspi praecox* can accumulate up to 80% Pb^49^ and Indian mustard (*Brassica junce*) has the tendency to accumulate upto 95% of total Pb^50^. Zhang^51^ conclude that rice cultivars different variable in metal uptake and translocation. Lead absorption and translocation to leaves depends on number of factors and these factors and variable among different crops. Shoot- accumulators store more lead in shoots of the plant while root-accumulator tends to store large amount of lead in roots and allow very small concentration of lead to be transported to above ground parts of crop. Another study revealed that uptake of lead by plants depends upon the surface area of roots^52^. Heavy metal effects root tip cells more as enlarged vacuole, disrupted cell membrane, plasmolysis and damaged mitochondrial thylakoid is more prominent in root cells^17^. Presence of different forms of lead in plants is also responsible for difference in its translocation rate. Sharma^53^ concluded that ions and lower molecular complexes are more mobile then other forms. Roots may absorb more lead in such plants but high molecular weight and complexes restrict its translocation and distribution to aerial crop part^54^. In an experiment conducted by Basharat et al., the lead induce reduction in *Brassica napus* L. biomass, reduced macronutrients in shoot, increased ROS and MDA^6^. Ali et al., reported the use of plant growth regulators (5-aminolevulinic acid, ALA) can cpounter the injurious effect of heavy metals in crops^15^.

**Table 6:** Accumulation of lead in shoots, roots and seeds of wheat (*Triticum aestivum* L.).

Treatments	Lead in shoots (μg/g)	Lead in roots (μg/g)	Lead in seeds (μg/g)
T_0_	0.9 ± 0.09 a	4.7 ± 0.05 a	0.194 ± 0.01 a
T1	15.6 ± 0.04 b	39.3 ± 0.03 b	0.213 ± 0.06 b
T_2_	22.4 ± 0.08 c	53.9 ± 0.09 c	0.271 ± 0.09 c
T_3_	37.3 ± 0.07 d	79.2 ± 0.03 d	0.304 ± 0.02 d
T_4_	49.1 ± 0.05 e	94.6 ± 0.01 e	0.356 ± 0.03 e
T_5_	60.7 ± 0.06 f	180.3 ± 0.05 f	0.383 ± 0.07 f
T_6_	87.3 ± 0.04 g	221.2 ± 0.02 g	0.422 ± 0.02 g
F-ratio	259,169.857 1	24.2617	22,153.4285
Significant level	***	***	***
LSD (*p* ≤ 0.05)	0.17512	1.4529	0.00175

T_0_ = 0 mg Pb/kg; T_1_ = 100 mg Pb/kg; T_2_ = 200 mg Pb/kg; T_3_ = 400 mg Pb/kg; T_4_ = 600 mg Pb/kg; T_5_ = 800 mg Pb/kg; T_6_ = 1,000 mg Pb/kg; Treatment means with different letters in the same column are significantly different from one another according to Duncan Multiple Range Test at (*p* ≤ 0.05); values represent the Means ± SE.

## Conclusion

The present study revealed that lead imparts number of negative effects on germination, physiological, biochemical, yield, mineral, and growth and quality attributes of wheat crop. It was observed that all adverse impacts of lead were due to reduced photosynthesis rate and related phenomenon like decreased rate of transpiration and stomatal conductance. Accumulation rate of lead in wheat parts (shoots, seeds and roots) was also above threshold level. Lead contamination in wheat is alarming as it is the staple food in Pakistan. Slow bioaccumulation of lead ultimately leads towards serious disorders and illness. And certain remedial techniques should be adopted to restrict the entry of lead from contaminated soils to wheat. Maintaining supply of lead free wheat is the need of an hour.

## Supplementary information

Supplementary file1
