# Supplementary material for: Effect of industrial wastewater on wheat germination, growth, yield, nutrients and bioaccumulation of lead
Source: Sci Rep. 2020 Jul 9;10:11361. doi: 10.1038/s41598-020-68208-7 (PMC7347546; doi:10.1038/s41598-020-68208-7)
Supplement: Supplementary file 1 — Supplementary file1 [file 41598_2020_68208_MOESM1_ESM.docx]

**Effect of industrial wastewater on wheat germination, growth, yield, nutrients and bioaccumulation of lead**

Amina Kanwal^1^, Muhammad Farhan^2,*^, Faiza Sharif^2^, Muhammad Umar Hayyat^2^, Laila Shahzad^2^, Gul Zareen^2^

*^1^Department of Botany, Government College Women University, Sialkot, Pakistan*

*^2^Sustainable Development Study Center, Government College University, Lahore, Pakistan*

^*^Corresponding Author: [m.farhan_gcu@yahoo.com](mailto:m.farhan_gcu@yahoo.com), +92 321 4122078

**Table S1 weather data during experimentation**

|  | **November 2017** | **December 2017** | **January 2017** | **February 2018** | **March**  **2018** | **April**  **2018** |
| --- | --- | --- | --- | --- | --- | --- |
| **Phonological stages** | Sowing,  Emergence | Tillering | shooting | Heading | Flowering  Milk maturity | Wax maturity  Full maturity  Harvest |
| **Average temperature (^o^C)** | 18 | 15 | 13 | 17 | 23 | 29 |
| **Maximum temperature (^o^C)** | 28 | 26 | 24 | 29 | 38 | 41 |
| **Minimum temparture (^o^C)** | 8 | 0 | 4 | 7 | 12 | 16 |
| **Relative humidity** | 53% | 63% | 68% | 57% | 52% | 46 |
| **Wind speed (km/h)** | 1 | 1 | 6 | 4 | 3 | 13 |
| **Average pressure (mbar)** | 1016 | 1018 | 1016 | 1016 | 1012 | 1008 |
| **Average sunlight Hours/day** | 8:42 | 7:05 | 6:50 | 7:57 | 7:40 | 9:38 |
| **Average daylight Hours and minutes /day** | 10:31 | 10:07 | 10:20 | 11:00 | 11:56 | 12:55 |
| **Sun altitude to solar noon on the 21^st^ day (^o^)** | 38.5 | 35 | 38.5 | 47.8 | 58.6 | 70.3 |
